# Vitamin D Intake in Slovenian Adolescents, Adults, and the Elderly Population

**DOI:** 10.3390/nu13103528

**Published:** 2021-10-08

**Authors:** Maša Hribar, Hristo Hristov, Živa Lavriša, Barbara Koroušić Seljak, Matej Gregorič, Urška Blaznik, Katja Žmitek, Igor Pravst

**Affiliations:** 1 Nutrition Institute, Tržaška Cesta 40, SI-1000 Ljubljana, Slovenia; masa.hribar@nutris.org (M.H.); hristo.hristov@nutris.org (H.H.); ziva.lavrisa@nutris.org (Ž.L.); katja.zmitek@vist.si (K.Ž.); 2 Biotechnical Faculty, University of Ljubljana, Jamnikarjeva 101, SI-1000 Ljubljana, Slovenia; 3 Computer Systems Department, Jozef Stefan Institute, SI-1000 Ljubljana, Slovenia; barbara.korousic@ijs.si; 4 National Institute of Public Health, Trubarjeva 2, SI-1000 Ljubljana, Slovenia; matej.gregoric@nijz.si (M.G.); urska.blaznik@nijz.si (U.B.); 5 VIST–Higher School of Applied Sciences, Gerbičeva cesta 51A, SI-1000 Ljubljana, Slovenia

**Keywords:** vitamin D, Slovenia, dietary intake, EU Menu, food propensity questionnaire, 24 h recall

## Abstract

Vitamin D is involved in calcium and phosphorus metabolism, and is vital for numerous bodily functions. In the absence of sufficient UV-B light-induced skin biosynthesis, dietary intake becomes the most important source of vitamin D. In the absence of biosynthesis, the recommended dietary vitamin D intake is 10–20 µg/day. Major contributors to dietary vitamin D intake are the few foods naturally containing vitamin D (i.e., fish), enriched foods, and supplements. The present study aimed to estimate the vitamin D intake in Slovenia, to identify food groups that notably contribute to vitamin D intake, and to predict the effects of hypothetical mandatory milk fortification. This study was conducted using data collected by the national cross-sectional food consumption survey (SI.Menu) in adolescents (*n* = 468; 10–17 years), adults (*n* = 364; 18–64 years), and the elderly (*n* = 416; 65–74 years). Data collection was carried out between March 2017 and April 2018 using the EU Menu Methodology, which included two 24-h recalls, and a food propensity questionnaire. Very low vitamin D intakes were found; many did not even meet the threshold for very low vitamin D intake (2.5 µg/day). Mean daily vitamin D intake was 2.7, 2.9, and 2.5 µg in adolescents, adults, and the elderly, respectively. Daily energy intake was found to be a significant predictor of vitamin D intake in all population groups. In adolescents and adults, sex was also found to be a significant predictor, with higher vitamin D intake in males. The study results explained the previously reported high prevalence of vitamin D deficiency in Slovenia. An efficient policy approach is required to address the risk of vitamin D deficiency, particularly in vulnerable populations.

## 1. Introduction

Vitamin D (VitD) deficiency and insufficiency are global health issues, posing a major public health risk [1,2]. Poor VitD status is connected with skeletal and non-skeletal health issues, including the functioning of the immune system [3,4,5,6,7]. Maintenance of an optimal VitD status is therefore of the utmost importance. The most common biomarker of VitD status is the serum concentration of 25-hydroxyvitamin D (25(OH)D). Optimal VitD status can be achieved by biosynthesis in the human skin when exposed to sufficient ultraviolet B light radiation (UVB) [8]. In the absence of or with insufficient UVB skin irradiation, VitD becomes an essential nutrient, and needs to be provided by dietary intake. Due to the changing solar zenith angle in European countries situated above the latitude of 35° N [9] (including Slovenia; 45–46° N [10]), the intensity of UVB light in wintertime is not sufficient to induce cutaneous synthesis of VitD [11]. In such cases, dietary intake becomes a major source of VitD [12,13]. The term *Vitamin D* usually covers two fat-soluble vitamers, namely ergocalciferol (D2) and cholecalciferol (D3). While D3 is obtained by endogenous synthesis via UVB exposure and diet (animal sources), the source of D2 is solely from the diet (from fungi). Overall, dietary VitD intake is obtained through food naturally rich in VitD, VitD-enriched food, or prescribed and over-the-counter medicines and supplements. The majority of foods are a naturally poor source of VitD, with the exception of oil-rich fish and eggs [12]. These VitD-rich foods tend to be consumed quite rarely or in smaller quantities [14], whereas poorer VitD sources (such as meat and meat products, milk products, and animal fats) are consumed regularly, and therefore constitute the majority of dietary VitD intake [15]. In addition to naturally occurring VitD, its content can be enhanced during production or processing; this is achieved through bioaddition and/or fortification. The term bioaddition is used to describe processes that are used during food production to enhance naturally occurring VitD, by feeding the animal a VitD-rich diet (used in meat and eggs) or by UVB irradiation of mushrooms or yeast [16]. The term fortification usually describes a process where either D2 or D3 is added near or at the end of food processing [16]. VitD fortification was initially used in cow’s milk to prevent rickets in Northern America and Europe [17]. In addition to dairy products, other types of foods are now also used as a vehicle of fortification, such as orange juice, cereal-based foods, and infant formulas [15]. Fortification can be either voluntary or mandatory policy. While a few countries, such as the USA, Canada, Australia, and Finland, have implemented regulated food fortification to increase dietary VitD intake [18], most countries have voluntary VitD fortification (also called “enrichment”) [18,19,20,21,22]. As in most European Union (EU) countries, this is also the case in Slovenia, where there are no recommendations regarding VitD fortification. The choice of fortification is in the hands of food manufacturers, and later is the choice of the end consumers. An important source of VitD intake is also supplementation with prescribed and over-the-counter medicines and food supplements. VitD is available both in multivitamin/multimineral and single-component products. These typically contain daily dosages of up to about 100 µg [16,23]. It should be noted that in Slovenia, VitD is routinely prescribed to children during the first year (10 µg), and further supplementation is recommended until the age of 18 years [24], while there is no official supplementation recommendation for the general adult population. However, in Slovenia during the COVID-19 pandemic, the typical non-medical prescribed VitD supplementation dosage in adults was 25 µg per day [23].

The low VitD status across the world is alarming [1,2,25,26], and this is also the case in Slovenia [11]. A previous study representative of Slovenia showed that during the extended winter period (November–April), 40.8% of adults in Slovenia had a serum 25(OH)D level below the critical level of 30 nmol/L, and 81.6% were below the recommended 50 nmol/L [11]; however, that study did not investigate dietary intake of VitD. It should be noted that thresholds for deficiency, insufficiency, and optimal status are still not fully harmonised across organisations [26,27,28,29,30,31], and there is a lack of consensus on the recommended daily intake of VitD [32]. The World Health Organisation (WHO) recommends 10 µg/day (400 International Units (IUs)) for those aged 51–65 years, and 15 µg/day (600 IU) for those aged over 65 years [27], whereas the daily recommended level by the European Food Safety Authority (EFSA) is 15 µg/day (600 IU) for all ages [30]. A level of 20 µg/day (800 IU) is recommended by D-A-CH (the nutrition societies of Germany, Austria, and Switzerland) [33]. These recommendations typically refer to VitD intake in the absence of UVB-induced endogenous synthesis [30,33]. Interestingly, for food labeling purposes, the nutrient reference value (NRV) for VitD is still set at 5 µg/day [34], and 50% of NRV (2.5 µg/day) has been used as a lower reference nutrient intake (LRNI) [35]. Several studies have reported VitD intakes well below the recommendations. In Europe, the reported VitD intake is generally between 3 and 5 µg per day [14,31,35,36,37,38,39,40], with higher intakes in Northern Europe (up to 11 µg/day) and lower in Southern Europe [14,26,37,38,41]. In Slovenia, only some specific population groups have been investigated (such as children [42,43], teenagers [44,45,46], and others [47,48,49,50,51]); nationally representative data for the healthy adult population are not available. Regarding the VitD intake, Lichthammer et al. [52] included populations (15–75 years) from four Central-Eastern European countries (including 81 subjects from Slovenia), and reported very low mean VitD intakes in all countries (the lowest in Austria with 2.2 µg daily, followed by Slovenia (2.6 µg), Poland (3.8 µg), and Hungary (4.1 µg)).

To address the challenge of the low VitD status in Slovenia, the *National expert working group on guidelines for sufficient vitamin D levels in the Slovenian population* was established by the National Institute of Public Health, at the request of the Ministry of Health of the Republic of Slovenia. The objective of the present study was to estimate the nationally representative VitD intake in the adolescent, adult, and elderly populations in Slovenia. We also aimed to identify food groups that notably contribute to VitD intake, and to estimate changes in dietary VitD intakes in the hypothetical scenario of mandatory milk fortification.

## 2. Materials and Methods

### 2.1. Study Design and Population

Data were collected within the scope of the cross-sectional Slovenian national food consumption survey (SI.Menu study). Data collection was carried out between March 2017 and April 2018 using the European Food Safety Agency (EFSA) Guidance on EU Menu Methodology [53]. The detailed study methodology is described in detail elsewhere [54]. In short, the participants were Slovenian residents, selected using the Central Register of the Population of Slovenia according to age, size and type of household, and place of residency. A total of 2280 individuals were allocated to three age groups: adolescents (10–17 years old), adults (18–64 years old), and the elderly (65–74 years old). Altogether, the response rate was 62.2% (*n* = 1319). The survey protocol was registered and accepted by the National Medical Ethics Committee (KME 0120-337/2016). All participants were informed about the details of the survey, and thereafter signed a written informed consent form. For the participants younger than 18 years of age, written consent was also obtained from the parent or legal guardian. Data were collected by skilled interviewers, during two interviews. The first interview included a general questionnaire, following by the food propensity questionnaire (FPQ), and the first 24 h dietary recall, while the second interview included second 24 h dietary recall. 

### 2.2. Dietary Assessment Methods

#### 2.2.1. General Questionnaire and Anthropometric Measurements

At the first face-to-face interview, participants completed a general questionnaire using computer-assisted personal interviewing. The questionnaire was adapted for adolescents and adults/the elderly. It included questions on socio-economic and socio-demographic determinants, such as marital status, place of living, level of education, employment status, and monthly income of the household. Participants also provided self-reported physical activity levels, which were converted to the International Physical Activity Questionnaire (IPAQ) score, as described by Craig et al. [55]. Participants’ weight and height were measured by using calibrated instruments at the end of the first interview.

#### 2.2.2. 24-h Dietary Recall

The interviewers performed two 24-h dietary recalls. The first recall was performed with a computer-assisted face-to-face interviewer, the second recall was repeated between 7 days to 3 weeks after the first one and was administered either by computer-assisted telephone interview or by face-to-face interview. During the recall, participants were asked to report their intake data for food and beverages consumed during the preceding day, following a daily meal timeline. Portion sizes were estimated using a nationally adjusted and validated picture book, developed in “Pilot study for the Assessment of Nutrient Intake and Food Consumption Among Kids in Europe” (PANCAKE), that contained 46 pictures of different food products or simple recipes, with each one photographed in six different portion sizes [54,56]. 

#### 2.2.3. Food Propensity Questionnaire

As recommended by the EFSA, a FPQ was used to record the usual frequency of consumption of specific foods and food supplements in the last 12 months [53,57]. In total, 75 food items were allocated into nine food groups: cereals and cereal products; milk and milk products; fruit; vegetables; meat, fish, eggs, and meat products; fats and fatty food; sugar and sweeteners; beverages; and miscellaneous. The frequency response options for the food list were never, 1–3 times per month or less, once per week, 2–3 times per week, 4–6 times per week, and 1–2 times per day or more. A special field was dedicated to food supplement use, where examples were listed (e.g., multivitamins, vitamin D, proteins, omega 3 and omega 6 fatty acids, etc.), and there was the possibility to add more. 

### 2.3. Assessment of Nutrient Intake

All foods and beverages reported during the 24-h recalls were assigned the appropriate energy and nutrient contents based on compositional data from the Open Platform for Clinical Nutrition (OPEN) [58]. The OPEN is a web-based application based on the national food composition database, which contains information and data for ingredients and recipes frequently used in Slovenia. To enable an accurate estimation of the nutritional composition of more complex foods and dishes, a disaggregation method was applied based on the recipes provided by the subjects, when applicable, or traditional recipes collected in the OPEN platform. To estimate the usual daily VitD intake in the population, we used the Multiple Source Method (MSM) analysis [59], in which reported foods were allotted into corresponding food categories, included in the FPQ [53].

All the extracted foods (*n* = 2377) from the SI.Menu consumption dataset were checked by a nutrition expert, and missing composition data were supplemented with VitD content. When the VitD content of the food was not found in OPEN, additional food composition databases were used (the National Food Composition Database in Finland (Fineli) [60], The Composition of Foods [61], or the United States Department of Agriculture Food Composition Database (USDA) [62]). Altogether, 63.4% of food items from the whole list were determined to be a source of dietary VitD. For assessment of the hypothetical VitD intake in the scenario of mandatory milk fortification, we assumed that all types of milk were enriched with an additional 2 µg of VitD per 100 mL [63]. 

### 2.4. Data Analysis

Exclusion criteria and assessment of under- and over-reporting subjects are explained in Zupanič et al. [64]. In short, subjects with incomplete anthropometric and/or 24-h recall data, and over-and under-reporting (based on the ratio of reported energy intake, with consideration of metabolic rate) were excluded. The final sample contained 1248 valid subjects: 468 adolescents, 364 adults, and 416 elderly subjects.

Usual VitD intake distributions per age group, adjusted for within individual day-to-day variation, were modelled with the MSM [59]. This method examines different food and nutrient distributions to estimate adjusted population distributions. It is characterised by a two-part shrinkage technique applied to residuals of two regression models, one for the positive daily intake data and one for the event of consumption. The shrunken residuals are back-transformed to their original scale, and the individual usual intake is obtained by multiplication of the frequency and amounts. The MSM was used to correct dietary intake data for intra- and inter-personal variability. Corrections were carried out only for food categories (28/101) identified as a source of VitD. Corrections for the reported frequency of food consumption were carried out for all categories which were included in the FPQ. We should note that while most (24/28) of the relevant food categories that are a source of VitD were included in the FPQ, this was not the case for a few products, including eggs. For such foods, the consumption was estimated only with consideration of 24 h recall data, without correction by FPQ data. Sex and body mass index (BMI) were included as covariates in the models. After the MSM was applied, individual usual daily intakes of VitD were calculated [65]. The same approach was used for the estimation of VitD intake in the hypothetical scenario of mandatory milk fortification, but different food composition data were used (details provided in Section 2.3).

Descriptive characteristics (mean, median, proportions) are presented for age cohorts and per different socio-demographic-, anthropometric-, and individual-based variables within each age group. Linear and logistic regression analyses were used to determine the significant differences between different sub-populations in terms of VitD intake. The adjusted means of VitD intake were determined by sex, place of living, BMI, and IPAQ levels for all age groups, while education and income were also used for adults and the elderly, and employment status was used only for adults. Some models were adjusted for energy intake. To report nationally representative epidemiological data, weighting was carried out with iterative proportional fitting [66], with consideration of age and sex, using census data from the 2017 reference population. The prevalence of very low VitD consumption was found using a previously defined LRNI threshold of 2.5 µg/day [35], separately for all age groups, with adjustments for socio-demographic, anthropometric, and lifestyle parameters. Model parameters were estimated by the maximum likelihood method. Odds ratios (ORs) with 95% confidence intervals (CIs) were used as a measure of relative risk for very low VitD intake (less than LRNI). Differences were considered significant at *p* < 0.05, except where it is stated otherwise.

The MSM online tool V1.0.1 (https://msm.dife.de/; accessed on 6 June 2021; the Department of Epidemiology of the German Institute of Human Nutrition Potsdam-Rehbrücke, Germany) was used for estimation of individual nutrient intakes, while statistical analyses were conducted using STATA V15.1 (StataCorp LLC, College Station, TX, USA). 

## 3. Results

We analyzed data for three population groups in the SI.Menu study [54]; adolescents, adults, and the elderly. The study population was representative for sex and age (10–17/18–64/65–75 years). The most relevant demographic and lifestyle characteristics of the study sample are outlined in Table 1. VitD supplementation was explicitly reported by 3.6%, 6.0%, and 4.8% of adolescents, adults, and the elderly, respectively. When considering both the use of VitD and multivitamin products, the proportion of supplementation was 17.1%, 17.3%, and 7.0%, in adolescents, adults, and the elderly, respectively (Table 1). Our study design unfortunately did not provide enough detail about supplementation patterns to estimate intakes of VitD with medicines and/or food supplements, or to investigate seasonal differences in supplementation practices. VitD intakes in this study, therefore, corresponded to consumption of regular foods, without food supplements.

A comparison of the 24 h recalls and FPQ data showed notable differences in the ability of both methods to identify true consumers of specific food categories (Supplementary Table S1). For example, the proportion of true consumers of sea fish was up to 10% higher when FPQ data were considered. Therefore, the usual dietary VitD food intakes were estimated using the MSM method, with consideration of both 24 h recalls and FPQ data [53].

Population-weighted dietary VitD intake for all three study populations is presented in Table 2. The mean VitD intake was 2.73 µg/day (95% CI: 2.56–2.91), 2.85 µg/day (95% CI: 2.69–3.00), and 2.45 µg/day (95% CI: 2.34–2.57) for adolescents, adults, and the elderly, respectively. When adjusting for energy intakes, mean VitD intakes were 1.24, 1.34 and 1.20 µg per 1000 kcal per day, respectively. The highest prevalence of very low VitD intakes (below 2.5 µg/day) was observed in the elderly population (61.0%), followed by adolescents (55.0%) and adults (45.8%). 

**Figure 1:**
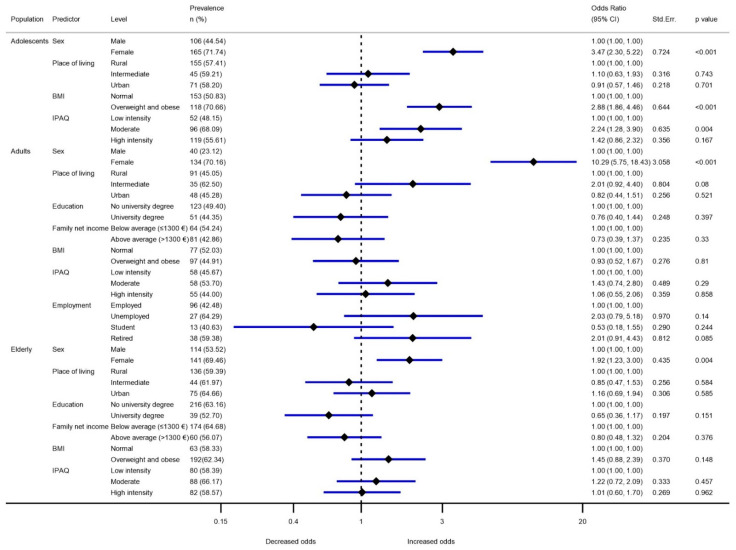
Percentage of the population with a very low vitamin D intake (lower reference nutrient intake; 2.5 µg/day) by sex, place of living, education, family net income, body mass index (BMI), International Physical Activity Questionnaire (IPAQ) score, and employment. **Notes**: BMI was considered to be normal when it was below 25 kg/m2, except for adolescents, where sex/age adjusted cut-off points [22,23] were used. Logistic regression analysis was conducted on samples with excluded missing values (Family net income: *n* = 57 (adults) and 40 (elderly); IPAQ: *n* = 5 (adolescents), 4 (adults), 6 (elderly)); Prevalence odds ratio for lower reference nutrient intake (<2.5 µg/day of vitamin D); lower reference nutrient intake prevalence probability test per different socio-demographic groups within age categories: *p* < 0.001 sex (adolescents), *p* < 0.001 BMI (adolescents), *p* < 0.05 IPAQ (adolescents); *p* < 0.001 sex (adults), *p* < 0.005 sex (elderly).

Adjusted mean VitD intakes by sex, place of living, BMI, IPAQ score, education, income, and employment for different age groups are presented in Table 3, with separate models for all three study populations, and are additionally adjusted for energy intake. Linear regression analyses showed that energy intake was a significant predictor of VitD intake for all three population groups (higher VitD intake for higher energy intake), while sex, education level, and BMI were significant predictors only in some models. Sex determined the VitD intake in adolescents and adults; in both cases, higher intakes were observed in males. Body mass index (BMI) was found to be a significant predictor only in adolescents, with lower VitD intake in those who were overweight/obese, while education was found to be a significant determinant of VitD intake in the elderly population, with the highest VitD intake in those with a university degree. Similar trends were also observed in other models investigating the likelihood of a very low VitD intake (Figure 1). For all three population groups, the model used a threshold of lower reference nutrient intake (LRNI; 2.5 µg/day). Sex was again found to be a significant predictor in adolescents (*p* < 0.001), adults (*p* < 0.001), and the elderly (*p* < 0.005), with higher odds ratios in females. In comparison with adolescent males, adolescent females were 3.47 (95% CI: 2.3–5.2) times more likely to have intakes below the LRNI, while in adults, this difference was even more pronounced (OR 10.29; 95% CI: 5.8–18.4). BMI was also a significant (*p* < 0.001) predictor in adolescents, with an OR 2.88 (95% CI: 1.9–4.5) times higher for overweight/obese subjects. 

**Figure 2:**
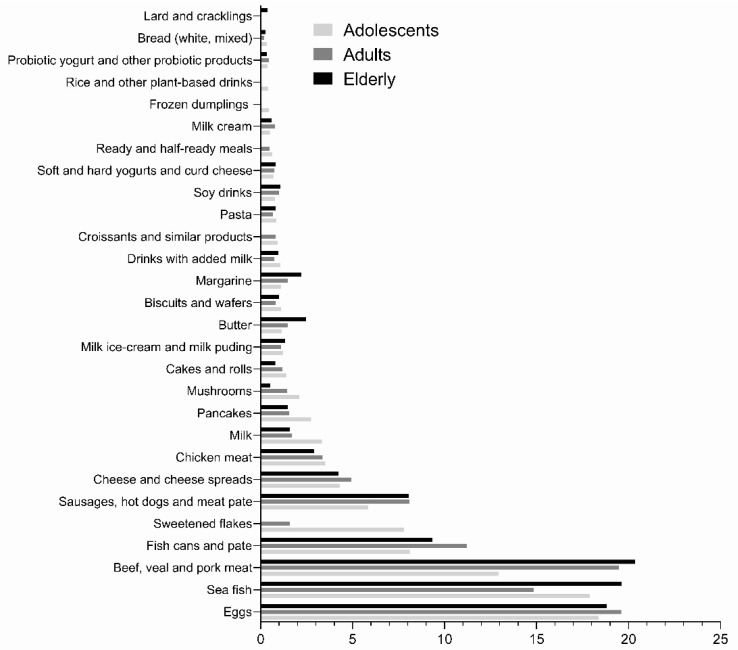
The relative contribution of food categories to Vitamin D intake among different population groups (% of total Vitamin D intake).

We further investigated the relative contributions of specific food categories to the dietary VitD intakes for the three investigated age groups (Figure 2). Beef, veal and pork meat, sea fish, and eggs were found to be the most important contributors of VitD intake among all age groups, with minor differences among groups. These food groups contributed from 13 to 20% of the dietary VitD intake. Processed meat and fish (fish cans and pate, and sausages, hot dogs, and meat pate) together contributed up to 11% of the VitD intake. In adolescents, processed breakfast cereals (sweetened flakes) were also found as a notable VitD source (~7%), while this was not observed in adults and the elderly population. 

For a more comprehensive insight into the importance of specific foods for VitD intake, we also investigated population-weighted consumption patterns for age groups (Supplementary Table S2), with the use of a threshold for LRNI (2.5 µg/day). In all age groups, those with a very low VitD intake reported a lower consumption of sea fish. In adolescents, this was also observed for beef, veal, and pork meat, sweetened flakes, and mushrooms. Among the adult population, those with intakes below LRNI had also lower intakes of fish cans and pate, sausages, hot dogs, and meat pate, milk, cakes and rolls, milk ice-cream and milk pudding, butter, biscuits and wafers, croissants and similar products, and ready and half-ready meals. In the elderly age group, the same applied to fish cans and pate, cheese and cheese products, milk, and mushrooms. 

**Figure 3:**
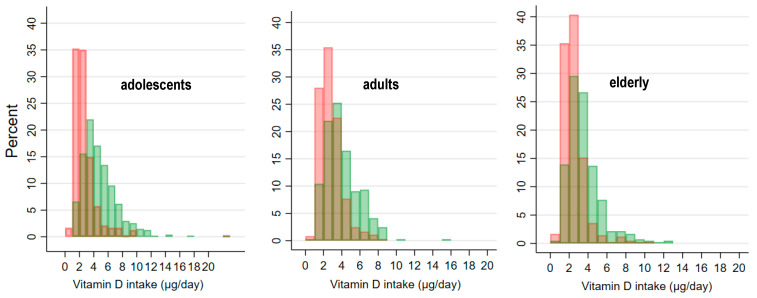
Histograms of vitamin D intakes from the regular diet (red bars) and in a projected fortified diet (scenario of mandatory fortification of milk with 2 µg Vitamin D per 100 mL) (green bars).

Our study also aimed to estimate changes in dietary VitD intakes in a hypothetical scenario of mandatory milk fortification. Using food intake data from all age groups, we modelled VitD intake in the scenario in which all milk would be fortified with 2 µg of VitD per 100 mL. As presented in Figure 3 and Table 4, the effect of such mandatory fortification would be most notable in adolescents. The projected increase in daily VitD intake due to milk fortification was 2 µg for adolescents and about 1 µg for adults and the elderly population. The estimated total mean VitD intake after accounting for the fortification would be 4.82 µg (SD: 2.51), 4.01 µg (SD: 1.88), and 3.58 µg (SD: 1.79) for adolescents, adults, and the elderly, respectively. 

## 4. Discussion

While a very high prevalence of VitD deficiency has been reported recently in Slovenia [11], there was no nationally representative data available on the dietary intakes of VitD. Using data collected by the Slovenian national food consumption survey (SI.Menu) we estimated daily VitD intakes using two non-consecutive 24 h dietary recalls and FPQ data. Three different age groups (adolescents, adults, and the elderly) were included in the analyses of VitD intake. That estimated VitD intakes were 2.73 µg/day, 2.85 µg/day, and 2.45 µg/day, respectively. While Lichthammer et al. [52] reported similar daily VitD intakes, they observed the highest intakes in their youngest age group (14–18 years; 3.72 µg/day). It should be noted that in their study, VitD intake was estimated with a different method (food frequency questionnaire; FFQ), and that their sample size was much smaller (*n* = 434 with 81 subjects from Slovenia). VitD intakes have been investigated in some other Slovenian populations, such as children [42,43], teenagers [44,45,46], and others [47,48,49,50,51]. VitD intake in adolescents varied from 2 to 4 µg/day [44,46], and was 2.1 µg/day in pregnant women [49]. In the elderly living in residential homes, intakes were particularly concerning (1 µg/day) [51]. When comparing our results to other European countries, we found many similarities. In two studies from neighboring Austria, the VitD intake was 2.1 µg/d (*n* = 4972; various dietary intake tools; all age groups above 4 years) [67], with the highest intakes in the adult population. The intake was 2.53 µg/d in a study by Kudlacek et al. (*n* = 1048; 21–76 years) [68]. In girls (11–17 years), the mean VitD intake ranged from 1.5 µg/day in Spain to 3.2 µg/day in Poland, whereas for boys (11–17 years), intakes ranged from 1.9 µg/day in France to 4.8 µg/day in Poland [35]. VitD intake in the elderly population (>65 years) ranged from 0.7 µg/day in Spain to 15 µg/day in Norway [38]. Across Europe, mean VitD intake varied from 1.1 to 6 µg per day [14,31,36,37,38,39,40], with higher intakes in Northern Europe (up to 14 µg/day) and lower intakes in Southern Europe [14,26,37,38,41]. Higher intakes in Northern Europe can be explained by the higher intake of fish and VitD enriched foods. 

In adolescents and adults, our results indicate higher intakes of VitD in males; however, this difference was not significant in the elderly population. The same pattern was observed when looking at the odds ratio (OR) for VitD intakes below the lower reference nutrient intake (LRNI; 2.5 µg/d). Adolescent males had an adjusted VitD intake of 2.95 µg/d, in comparison to 2.52 µg/d in females. Meanwhile, the adjusted intake in adult males was 3.21 µg/d in comparison to 2.31 µg/d in adult females. Males usually have higher VitD intakes than women [14,37], which is also related to the higher amount of consumed food. This was also observed in our study, where daily energy intake was a significant predictor of VitD intake. Furthermore, in adolescents, those with normal BMI had a significantly higher intake of VitD (and lower OR for very low VitD intake) in comparison with overweight or obese adolescents. Similar observations were made in a study from Northern Norway [69], but in the adult population. In the elderly, VitD intake was statistically higher among those with a higher education level. Similar observations were reported in two Swiss studies in some population groups [70,71]. 

To investigate the prevalence of very low VitD intake, we took the cut-off value of 2.5 µg/day. The prevalence of intake below the LRNI was 55.0, 45.8, and 61.0% in adolescents, adults, and the elderly, respectively. The highest prevalence of below LRNI intake was among teenage females (72.6%), and the lowest was among adult males (21.0%). In the population of adolescent males, 38.8% had intakes below 2.5 µg/day, in comparison to 72.6% of females. In European adolescents (11–17 years), the prevalence of intakes of VitD below the LRNI ranged from 17.1% (Netherlands) to 81.7% (France) in males, and 36.6% (Netherlands) to 97.9% in females (Spain) [35]. In the Slovenian adult population, males had a much lower prevalence of below LRNI intakes (21.0%) than females (71.0%). The prevalence data from other European countries were also very varied, ranging from 26.8% (Netherlands; 31–60 years) to 94.7% (Spain; 18–60 years) in adult females, and from 7.3% (Netherlands; 31–60 years) to 87.4% (Spain; 18–60 years) in adult males [35]. In our elderly population, 58.2% of males and 63.5% of females had below LRNI intakes. In other elderly European populations (<60 years) [35], the prevalence ranged from 17.4% (Netherlands) to 100% (Spain) in females, and from 5.7% (Netherlands) to 100% (Spain) in males. We should also note that a notable trend of lower risk for very low VitD intake was observed in those with higher financial status in both adults and elderlies (OR 0.73 and 0.80, respectively), but was not statistically significant. This also corresponded to the observed trend of increased OR for unemployed (OR 2.03, *p*=0.14).

In the present study, the main contributors to VitD intake across age groups were eggs, sea fish, beef, veal, and pork, fish cans and pate, sweetened flakes (only in adolescents), and sausages, hot dogs, and meat pate. Likewise, in other European countries, the leading food group contributors to VitD intake were fish/shellfish, added fats, meats/meat products, cakes, cereals, and dairy products [31,37,72].

Very low dietary intake of VitD in the Slovenian population, shown in our study, partially explains the previously reported high prevalence of VitD deficiency during winter, when sun-induced biosynthesis of VitD is not sufficient [11]. While the public health outcomes of this epidemiological situation have not been well investigated, it should be mentioned that VitD is a key component in various bodily functions [3,4,5,6,7], also related to the functioning of the immune system and bone health. Although this study did not address this topic, it should be mentioned that Slovenia has been among the countries with the most severe death toll from COVID-19 (currently 2,174 deaths/1 mio. population [73]; the majority of those deaths occurred during the autumn/winter pandemic wave in 2020/21), and that we also have quite a high prevalence of osteoporosis in comparison with some other European countries. For example, in Slovenia, 27.5% of women over 50 years had osteoporosis [74], while reported rates for France and Span are much below 20% [75].

In Slovenia, there is no mandatory fortification of foods with VitD, or even national recommendations. However, rules for the enrichment of foods are defined in the EU Regulation No 1925/2006 on the addition of vitamins and minerals and of certain other substances to foods [21], which enables the enrichment of foods with VitD using either cholecalciferol or ergocalciferol [76]. The fortification of foods with VitD (either mandatory or voluntarily based on national recommendations) is currently in place in North American and some European countries [77,78,79]. The amount of added VitD varies across countries, as do the food matrixes. Typical matrixes for fortification include milk and dairy products. In countries with implemented VitD fortification policies, the amount of added VitD is increasing [79]. Canada recently proposed a mandatory policy to increase fluid milk fortification from 1 µg to 2 µg/100 mL, due to inadequate VitD intake in the population [63]. Our goal was to investigate a hypothetical scenario in which milk was fortified with 2 µg of VitD per 100 mL of milk, as proposed in Canada [63]. The projected increase in daily dietary VitD intake in this model was 2.0 µg, 1.1 µg, and 1.0 µg in adolescents, adults, and the elderly, respectively. It should be noted that a review of Black et al. [80] showed that each ingested microgram of VitD with fortified food leads to an increase in serum 25(OH)D of 1.2 nmol/L. However, the problem of limiting fortification to milk is in the fact that not everyone in the population consumes milk (i.e., due to lactose intolerance, etc.), and the idea of mandatory fortification is to protect the general population. A preferable fortification approach may therefore be adding VitD in smaller amounts to various food matrixes [81]. Jääskeläinen et al. [77] reported the effects of the implementation of VitD fortification on VitD status in Finland. Their approach was to fortify both fluid milk (1.0 µg/100 g) and spreadable fats (20 µg/100 g). The intervention resulted in higher VitD intakes and a lower prevalence of VitD deficiency, based on serum 25(OH)D levels [77]. We should also mention that in Finland and the United States, fortified foods have the highest contribution to dietary VitD intake [82]. 

The strength of our study is that VitD intake was estimated with the exploitation of data collected with the Slovenian national SI.Menu food consumption survey, using both 24 h recalls and food propensity questionnaire data. Another strength is that sampling was carried out with three quotas, enabling insights into the more vulnerable populations of adolescents and the elderly. We should also mention that since the SI.Menu study collected a series of sociodemographic and lifestyle indicators, we were able to include those in the regression analyses. Study limitations should be also noted. While the SI.Menu study was designed using a very robust EU Menu methodology and harmonized with the EFSA, the study was not primarily designed to investigate dietary VitD intakes. The objective of the SI.Menu study was to collect data on food consumption in order to inform regulatory risk assessments related to the use of food additives, food contaminants, etc. Nutritional assessment was a secondary objective, and the FPQ was therefore not tailored for this purpose. Because of this, our FPQ dataset did not include the frequency of consumption of some foods (particularly eggs), which are an important source of VitD in the diet. Considering the study design, food composition (VitD content) needed to be estimated using food composition databases, and not with laboratory analyses. A methodological limitation is that in Slovenia, national food composition data do not include the amount of VitD in some foods, and we were therefore forced to use additional food composition databases. An important study limitation is also that we could only estimate daily intake of VitD with foods, without accounting for the use of medicines and food supplements. While the consumption of such products was included in the FPQ of the SI.Menu study, the collected data did not enable assessment of the corresponding VitD intake. The only available data regarding VitD supplements was if a person was taking them. In the present study, 3.6%, 6.0%, and 4.8% of adolescents, adults, and the elderly, respectively, reported the use of VitD supplements. However, supplementation can be an important additional source of VitD [14,83]. For example, a typical dosage of VitD in food supplements is much higher than the expected intake with foods, at 25 µg VitD daily dosage [23]. Furthermore, the COVID-19 pandemic changed people’s behaviors considerably [84], and a much higher frequency of VitD supplementation was reported during the 2020 COVID-19 lockdown in Slovenia [85], affecting VitD intakes considerably. However, the long-term effects of the pandemic are not known. Ideally, further research should focus on the assessment of VitD intakes from all sources in the post-COVID-19 period.

## 5. Conclusions

The estimated dietary intake of VitD in the Slovenian population was very low; 2.73 µg, 2.85 µg, and 2.45 µg in adolescents, adults, and the elderlies, respectively. The VitD intake was well below the recommended intake in all age groups. Most of the population did not even meet the threshold for a lower reference nutrient intake (LRNI; 2.5 µg/day). In all population groups, daily energy intake was a significant predictor of VitD intake. An additional predictor for adolescents and adults was also sex, with higher VitD intakes among males. Higher BMI was also found to be a predictor for lower VitD intake in adolescents, while in the elderly population this was observed in those with lower education levels. The main contributors to VitD intake in the Slovenian diet were eggs, fish and fish products, and meat and meat products. Assessment of the hypothetical scenario of mandatory milk fortification with 2 µg VitD per 100 mL showed a notable increase in the predicted VitD intake, with the highest effect in adolescents, but the expected dietary VitD intake would still be notably below the recommended intake. 

## Figures and Tables

**Table 1:** Demographic and lifestyle characteristics of the study sample.

		Age Cohorts		
		Adolescents	Adults	Elderly
		(10–17 Years)	(18–64 Years)	(65–74 Years)
		*n* = 468	*n* = 364	*n* = 416
Age; years—mean (SD)		13.4 (2.37)	43.6 (13.81)	68.7 (2.7)
Place of living—*n* (%)	Rural	270 (57.7)	202 (55.5)	229 (55.1)
	Intermediate	76 (16.2)	56 (15.4)	71 (17.1)
	Urban	122 (26.1)	106 (29.1)	116 (27.9)
Sex—*n* (%)	Male	238 (50.9)	173 (47.5)	213 (51.2)
	Female	230 (49.1)	191 (52.5)	203 (48.8)
Education—*n* (%)	No university degree	n.a.	249 (68.4)	342 (82.2)
	University degree	n.a.	115 (31.6)	74 (17.8)
Family monthly net income—*n* (%)	Below average	n.a.	118 (38.4)	269 (71.5)
	Above average	n.a.	189 (61.6)	107 (28.5)
BMI—mean (SD)		21.0 (4.2)	26.7 (5.2)	28.4 (5.0)
*n* (%)	Normal	301 (64.6)	148 (40.7)	108 (26.0)
	Overweight and obese	167 (35.7)	216 (59.3)	308 (74.0)
IPAQ—*n* (%)	Low intensity	108 (23.3)	127 (35.3)	137 (33.4)
	Moderate	141 (30.5)	108 (30.0)	133 (32.4)
	High intensity	214 (46.2)	125 (34.7)	140 (34.2)
Employment status—*n* (%)	Employed	n.a.	226 (62.1)	n.a.
	Unemployed	n.a.	42 (11.5)	n.a.
	Student	n.a.	32 (8.8)	n.a.
	Retired	n.a.	64 (17.6)	n.a.
Use of dietary supplements—*n* (%)	Vitamin D	17 (3.63)	22 (6.04)	20 (4.81)
	Multivitamin	72 (15.4)	52 (14.3)	11 (2.64)
	Vitamin D and/or multivitamin supplements	80 (17.1)	63 (17.3)	29 (6.97)

**Notes**: Body mass index (BMI) was considered to be normal when it was below 25 kg/m^2^, except for adolescents, where sex/age adjusted cut-off points [22,23] were used; International Physical Activity Questionnaire (IPAQ); standard deviation (SD); not applicable (n.a).

**Table 2:** Population-weighted dietary vitamin D intake (µg/day), and prevalence of very low vitamin D intake.

	Adolescents (10–17)			Adults (18–64)			Elderly (65–74)		
	All	Male	Female	All	Male	Female	All	Male	Female
**Sample Size, *n* (%)**	**468 (100)**	238 (50.85)	230 (49.15)	**364 (100)**	173 (47.53)	191 (52.47)	**416 (100)**	213 (51.20)	203 (48.80)
Vitamin D intake									
Mean [µg/day] (95% CI)	**2.73** **(2.56–2.91)**	3.02 (2.83–3.22)	2.42 (2.14–2.70)	**2.85** **(2.69–3.00)**	3.39 (3.17–3.62)	2.30 (2.14–2.44)	**2.45** **(2.34–2.57)**	2.60 (2.42–2.78)	2.32 (2.16–2.48)
Std.Err.	**0.09**	0.10	0.14	**0.08**	0.12	0.08	**0.06**	0.09	0.08
Median [µg/day]	**2.33**	2.70	1.95	**2.66**	3.01	2.04	**2.21**	2.30	2.13
Mean [µg/1000 kcal per day] (95% CI)	**1.24** **(1.15–1.33)**	1.23 (1.12–1.35)	1.25 (1.11–1.40)	**1.34** **(1.27–1.41)**	1.47 (1.37–1.57)	1.21 (1.13–1.29)	**1.20** **(1.13–1.27)**	1.20 (1.10–1.30)	1.20 (1.10–1.29)
Prevalence of very low vitamin D intake (%)									
<2.5 [µg/day]	**55.0** **(47.4–62.4)**	38.8 (30.0–48.5)	72.6 (64.6–79.3)	**45.8** **(40.1–51.6)**	21.0 (15.2–28.2)	71.0 (63.5–77.5)	**61.0** **(51.7–69.6)**	58.2 (43.6–71.5)	63.5 (50.6–74.7)

**Notes**: 95% CI: 95% confidence interval.

**Table 3:** Unadjusted and adjusted mean (95% CI) levels of vitamin D intake (µg/day) by sex, place of living, body mass index (BMI), International Physical Activity Questionnaire (IPAQ) score, education, income, employment, and energy intake (kcal/day) for different age groups. All three models were adjusted to account for differences in energy intake (kcal/day).

Variable		Adolescents (10–17 Years Old)		Adults (18–64 Years Old)		Elderly (65–74 Years Old)	
		Unadjusted	Adjusted	Unadjusted	Adjusted	Unadjusted	Adjusted
**Sex**	**Male**	3.01 (2.82–3.20)	2.95 (2.72–3.17)	3.35 (3.16–3.53)	3.22 (3.03–3.42)	2.67 (2.51–2.83)	2.55 (2.37–2.73)
	**Female**	2.44 (2.18–2.70)	2.52 (2.29–2.76)	2.31 (2.15–2.46)	2.46 (2.28–2.64)	2.35 (2.16–2.54)	2.49 (2.30–2.68)
**Place of living**	**Rural**	2.64 (2.46–2.80)	2.63 (2.42–2.84)	2.88 (2.71–3.05)	2.86 (2.68–3.03)	2.57 (2.41–2.73)	2.56 (2.39–2.73)
	**Intermediate**	2.87 (2.24-3.50)	2.85 (2.46-3.24)	2.66 (2.25–3.07)	2.68 (2.36–3.00)	2.38 (2.06–2.71)	2.50 (2.20–2.81)
	**Urban**	2.86 (2.54–3.17)	2.90 (2.60–3.21)	2.73 (2.52–2.94)	2.81 (2.59–3.04)	2.48 (2.24–2.72)	2.44 (2.20–2.69)
**Education**	**No university degree**	n.a.	n.a.	2.74 (2.59–2.88)	2.76 (2.61–2.92)	2.43 (2.31–2.55)	2.45 (2.31–2.60)
	**University degree**			2.94 (2.69–3.19)	2.92 (2.69–3.16)	2.88 (2.47–3.29)	2.85 (2.52–3.17)
**Family net income**	**Below average (≤EUR 1300)**	n.a.	n.a.	2.63 (2.40–2.85)	2.71 (2.50–2.93)	2.44 (2.30–2.60)	2.51 (2.33–2.64)
	**Above average (>EUR 1300)**			2.92 (2.74–3.10)	2.89 (2.71–3.05)	2.68 (2.42–2.94)	2.56 (2.31–2.81)
**BMI**	**Normal**	2.95 (2.72–3.16)	2.94 (2.75–3.14)	2.72 (2.52–2.92)	2.81 (2.60–3.01)	2.51 (2.28–2.73)	2.57 (2.31–2.83)
	**Overweight and obese**	2.34 (2.13–2.56)	2.35 (2.10–2.62)	2.86 (2.69–3.02)	2.82 (2.66–2.98)	2.52 (2.37–2.66)	2.50 (2.36–2.65)
**IPAQ**	**Low intensity**	2.77 (2.55–2.99)	2.72 (2.40–3.05)	2.79 (2.60–2.99)	2.78 (2.56–2.99)	2.56 (2.33–2.78)	2.54 (2.32–2.76)
	**Moderate**	2.64 (2.27–3.02)	2.71 (2.42–3.00)	2.74 (2.48–2.98)	2.82 (2.60–3.05)	2.30 (2.14–2.46)	2.35 (2.13–2.57)
	**High intensity**	2.79 (2.56–3.01)	2.77 (2.53–2.99)	2.89 (2.65–3.12	2.85 (2.63–3.06)	2.69 (2.45–2.93)	2.67 (2.45–2.89)
**Employment**	**Employed**	n.a.	n.a.	2.91 (2.75–3.08)	2.81 (2.64–2.98)	n.a.	n.a.
	**Unemployed**			2.51 (2.14–2.87)	2.85 (2.46–3.23)		
	**Student**			2.91 (2.53–3.29)	2.96 (2.47–3.44)		
	**Retired**			2.54 (2.23–2.86)	2.76 (2.44–3.07)		

**Notes:** BMI was considered to be normal when it was below 25 kg/m^2^, except for adolescents, where sex/age adjusted cut-off points [22,23] were used. Linear regression analysis conducted on samples with excluded missing values (Family net income: *n* = 57 (adults) and 40 (elderly); IPAQ: *n* = 5 (adolescents), 4 (adults), 6 (elderly)); Difference in predicted marginal means of vitamin D intake per different socio-demographic groups within age categories: *p* < 0.05 sex (adolescents), *p* < 0.001 BMI (adolescents); *p* < 0.0001 sex (adults); *p* < 0.05 education (elderly). The energy intake was a significant predictor of vitamin D intake, with β = regression coefficients per different age group as follows: β = +0.0004 (*p* ≤ 0.001) for adolescents, β = +0.0006 (*p* ≤ 0.001) for adults, β = +0.0006 (*p* ≤ 0.001) for the elderly.

**Table 4:** Comparison of estimated vitamin D intake (μg/day) from a regular diet and projected fortified diet.

	Adolescents (10–17)			Adults (18–64)			Elderly (65–74)		
	All	Male	Female	All	Male	Female	All	Male	Female
Unadjusted mean vitamin D intake—μg/day (SD)									
Regular diet	2.73 (1.77)	3.01 (2.44)	2.44 (2.00)	2.80 (1.24)	3.35 (1.23)	2.31 (1.03)	2.51 (1.26)	2.67 (1.16)	2.35 (1.35)
Projected fortified diet *	4.82 (2.51)	5.46 (2.29)	4.16 (2.56)	4.01 (1.88)	4.53 (1.90)	3.56 (1.73)	3.58 (1.79)	3.62 (1.83)	3.54 (1.75)
Median vitamin D intake—μg/day									
Regular diet	2.25	2.66	1.95	2.59	3.02	2.02	2.26	2.38	2.01
Projected increase with fortification *	2.01	4.10	3.15	1.10	1.04	1.23	1.01	0.87	1.25
Projected fortified diet *	4.26	6.76	5.1	3.69	4.06	3.25	3.27	3.25	3.26

**Notes**: Vitamin D intakes in the study sample, without population weighting. SD: Standard deviation; * Projected fortified diet with the scenario of mandatory fortification of milk with 2 µg Vitamin D per 100 mL.

## Data Availability

The data presented in this study are available on request from the corresponding author.

## Supplementary Materials

The following are available online at https://www.mdpi.com/article/10.3390/nu13103528/s1, Supplementary Table S1. Patterns of observed food consumption related to vitamin D in the diet: comparison of true consumers with the use of FPQ data and 24 h dietary recall. Supplementary Table S2. Patterns of observed FPQ food consumption related to vitamin D in the diet in subjects with intakes lower and higher than 2.5 µg vitamin D per day.
