# Supplementary material for: Vitamin D Intake in Slovenian Adolescents, Adults, and the Elderly Population
Source: Nutrients. 2021 Oct 8;13(10):3528. doi: 10.3390/nu13103528 (PMC8537619; doi:10.3390/nu13103528)
Supplement: Supplementary file 1 [file nutrients-13-03528-s001.zip › nutrients-1396463-supplementary.pdf]

Supplementary Table S1. Patterns of observed food consumption related to vitamin D diet: comparison of true consumers with the use of FPQ data and 24-h dietary recalls

Food categories	Adolescents (10–17 Years Old)		Adults (18–64 Years Old)		Elderly (65–74 Years Old)	
	True consumers FPQ (%)	Consumers in 24 h dietary recalls (%)	True consumers FPQ (%)	Consumers in 24 h dietary recalls (%)	True consumers FPQ (%)	Consumers in 24 h dietary recalls (%)
**Sea fish**	75.8	65.4	80.8	72.8	73.8	68.8
**Beef, veal and pork meat**	91.5	98.3	95.3	96.4	96.6	99.3
**Fish cans and pate**	73.1	67.5	75.6	66.8	72.1	71.4
**Sweetened flakes**	68.2	69.4	24.2	22.5	8.17	1.20
**Sausages, hot dogs and meat pate**	94.2	95.3	86.3	87.9	88.9	90.9
**Cheese and cheese spreads**	88.9	95.5	91.5	94.0	90.1	91.4
**Chicken meat**	98.1	98.3	96.4	97.0	97.8	98.3
**Milk**	92.7	96.6	82.7	85.7	83.7	87.5
**Mushrooms**	57.7	64.7	77.2	79.4	79.6	73.8
**Cakes and rolls**	89.5	84.0	80.8	58.0	73.0	46.4
**Milk ice-cream and milk pudding**	93.8	80.6	75.8	37.1	63.5	7.69
**Butter**	85.3	85.9	82.1	85.4	83.9	86.3
**Biscuits and wafers**	94.2	90.4	81.3	82.1	72.8	72.8
**Margarine**	54.0	65.8	53.2	53.0	45.2	48.6
**Croissants and similar products**	90.8	92.1	82.9	51.7	80.1	0.0
**Pasta**	99.2	94.7	96.7	98.4	96.9	95.2
**Soya drinks**	12.2	7.26	7.42	3.57	4.81	1.92
**Soft and hard yogurts and curd cheese**	90.8	92.1	92.0	91.8	90.4	90.1
**Ready and half-ready meals**	55.3	51.9	47.3	46.4	28.7	0.00
**Eggs***		98.5		100		100
**Pancakes***		99.8		87.9		97.1
**Lard and cracklings**	57.9	41.0	71.2	45.9	78.1	31.7
**Probiotic yogurts and other probiotic products**	44.6	39.7	48.6	34.3	28.7	28.2
**Drinks with added milk***		25.4		77.5		70.0
**Rise and other plant-based drinks**	13.7	3.63	11.3	0.55	5.5	0
**White and halfwhite bread**	95.3	70.7	84.9	15.9	76.7	27.6
**Mixed and whole wheat bread**	85.5	50.2	88.5	0.00	89.7	0.00
**Milk (cream)***		94.7		86.8		97.6

Notes: FPQ: food propensity questionnaire. *Food groups for which FPQ data were not collected.

Supplementary Table S2. Patterns of observed FPQ food consumption related to vitamin D diet in subjects with intakes lower and higher than 2.5 μg vitamin D per day

FFQ categories	Proportions of food consumers based on FPQ data; % (95% CI)								
	Adolescents (10-17)			Adults (18-64)			Elderly (65-74)		
	All	<2.5 μg/day	>2.5 μg/day	All	<2.5 μg/day	>2.5 μg/day	All	<2.5 μg/day	>2.5 μg/day
**Sea fish**	78.5 (73.5-82.8)	72.7 (65.7-78.7)	85.6 (78.5-90.6)	83.1(78.6-86.7)	74.5 (67.0-80.8)	90.3 (84.9-93.9)	78.8 (73.0-83.6)	72.3 (62.8-80.0)	89.0 (82.8-93.1)
**Beef, veal, and pork meat**	92.8 (89.9-94-9)	89.6 (85.0-92.9)	96.7 (93.1-98-4)	95.1 (92.2-97.0)	92.8 (87.6-95.9)	97.1 (92.8-98.8)	97.4 (95.4-98.5)	96.3 (93.1-98.1)	99.0 (96.2-99.8)
**Fish cans and pate**	75.4 (65.0-80.1)	71.6 (64.5-77.7)	80.0 (71.4-86.5)	77.9 (73.0-82.2)	69.9 (61.9-76.8)	84.7 (78.5-89.4)	71.5 (62.79-79.0)	65.7 (54.8-75.2)	80.7 (59.1-92.3)
**Sweetened flakes**	70.9 (65.2-76.1)	63.9 (56.2-71.1)	79.5 (71.1-85.9)	25.6 (20.8-31.1)	24.8 (18.2-32.8)	26.3 (19.8-34.2)	7.6 (4.9-11.9)	9.9 (5.6-16.8)	4.1 (2.0-8.1)
**Sausages, hot dogs, and meat pate**	94.6 (91.4-96.7)	96.5 (93.7-98.1)	92.2 (85.5-96.0)	85.9(81.5-89.3)	79.6 (72.1-85.4)	91.2 (85.7-94.7)	89.7 (85.6-92.7)	86.5 (80.1-91.1)	94.6 (89.3-97.3)
**Cheese and cheese spreads**	86.5 (78.4-91.9)	83.8 (70.1-91.9)	89.9 (82.5-94.4)	90.9 (86.6-93.9)	88.2 (81.7-92.6)	93.2 (86.5-96.7)	91.5 (87.9-94.2)	88.0 (82.0-92.3)	97.0 (93.3-98.7)
**Chicken meat**	98.2 (96.4-99.1)	97.9 (95.0-99.2)	98.5 (95.8-99.5)	96.9 (93.8-97.9)	95.3 (90.7-97.7)	97.3 (93.6-98.9)	97.1 (92.4-99.0)	95.6 (87.9-98.5)	99.6 (97.0-99.9)
**Milk**	94.3 (91.2-96.2)	94.1 (90.4-96.4)	94.6 (89.9-97.2)	84.4 (79.9-88.0)	80.3 (73.0-86.1)	87.7 (82.0-91.8)	75.2 (60.4-85.8)	71.8 (51.4-86.0)	80.6 (59.2-92.2)
**Mushrooms**	57.3 (50.0-64.4)	49.1 (40.5-57.6)	67.5 (57.2-76.3)	76.4 (71.0-81.1)	73.2 (64.1-80.1)	79.2 (71.9-85.0)	73.3 (60.0-83.4)	69.0 (50.0-83.2)	80.0 (66.8-88.8)
**Cakes and rolls**	89.8 (85.9-92.7)	89.9 (85.2-93.2)	89.8 (82.6-94.2)	81.2 (76.4-85.2)	75.6 (67.7-82.0)	85.9 (79.8-90.4)	68.3 (55.5-78.9)	66.7 (48.6-80.9)	70.9 (53.7-83.7)
**Milk ice-cream and milk pudding**	95.3 (93.1-96.7)	94.4 (91.0-96.6)	96.5 (93.3-98.2)	77.7 (72.7-82.0)	71.0 (63.0-78.0)	83.3 (77.2-88.1)	58.2 (47.2-68.5)	58.0 (42.8-71.8)	58.6 (43.5-72.2)
**Butter**	84.4 (79.4-88.4)	82.8 (75.9-88.0)	86.5 (78.3-91.9)	82.3 (77.6-86.2)	78.6 (71.0-84.7)	85.4 (79.2-90.0)	87.2 (82.9-90.6)	85.0 (78.5-89.8)	90.6 (84.4-94.5)
**Biscuits and wafers**	95.2 (92.9-96.8)	94.5 (91.0-96.8)	96.1 (92.4-98.0)	82.1 (77.3-86.0)	77.6 (69.9-83.7)	85.9 (79.6-90.4)	77.1 (71.0-82.2)	75.7 (67.0-82.7)	79.3 (70.6-85.9)
**Margarine**	53.6 (46.3-60.8)	50.9 (42.2-59.6)	56.9 (45.4-67.8)	48.3 (42.5-54.1)	44.8 (36.7-53.3)	51.2 (43.2-59.1)	41.8 (33.4-50.6)	43.8 (32.1-56.3)	38.5 (28.0-50.2)
**Croissants and similar products**	90.2 (85.8-93.4)	89.1 (82.6-93.3)	91.6 (84.7-95.6)	84.4 (80.0-88.0)	78.2 (70.8-84.2)	89.6 (84.1-93.4)	83.8 (78.9-87.8)	81.3 (73.8-87.0)	87.9 (81.1-92.4)
**Pasta**	99.4 (98.4-99.8)	99.6 (98.2-99.9)	99.3 (96.2-99.9)	96.6 (93.6-98.2)	96.3 (90.9-98.6)	96.8 (92.7-98.6)	97.4 (95.2-98.6)	97.1 (94.0-98.6)	97.8 (93.5-99.3)
**Soya drinks**	11.8 (8.5-16.1)	12.4 (7.9-18.9)	11.13 (6.95-17.4)	7.5 (4.97-.11.1)	7.04 (3.68-13.1)	7.85 (4.6-13.0)	4.07 (2.47-6.63)	2.70 (1.22-5.86)	6.21 (3.28-11.5)
**Soft and hard yogurts and curd cheese**	91.2 (87.8-93.8)	90.2 (85.5-93.4)	92.5 (86.6-95.9)	92.6 (89.2-94.9)	88.5 (82.5-92.7)	96.0 (91.8-98.1)	93.6 (90.7-95.6)	93.5 (89.8-95.9)	93.6 (88.0-96.7)
**Ready and half-ready meals**	56.2 (48.9-63.2)	51.4 (42.6-60.1)	62.0 (51.1-71.8)	49.8 (44.0-55.6)	44.1 (35.8-52.8)	54.7 (46.7-62.5)	30.1 (23.1-38.2)	27.9 (19.5-38.2)	33.6 (22.7-46.7)

Notes: FPQ: food propensity questionnaire
